# Pharmacokinetics of UGN-101, a mitomycin-containing reverse thermal gel instilled via retrograde catheter for the treatment of low-grade upper tract urothelial carcinoma

**DOI:** 10.1007/s00280-021-04246-w

**Published:** 2021-03-07

**Authors:** Ahmad Shabsigh, Nir Kleinmann, Angela B. Smith, Douglas Scherr, Elyse Seltzer, Mark Schoenberg, Seth P. Lerner

**Affiliations:** 1 Department of Urology, The Ohio State University Comprehensive Cancer Center, 300 W 10th Ave, Columbus, OH 43210 USA; 2 Department of Urology, Sheba Medical Center, Ramat Gan, Israel; 3 Department of Urology, University of North Carolina School of Medicine, Chapel Hill, NC USA; 4 Department of Urology, Weill Medical College of Cornell University, New York, NY USA; 5 UroGen Pharma, New York, NY USA; 6 Department of Urology, Albert Einstein College of Medicine, Bronx, NY USA; 7 Scott Department of Urology, Baylor College of Medicine, Houston, TX USA

**Keywords:** UGN-101, Mitomycin, Pharmacokinetics, Upper tract urothelial carcinoma, UTUC

## Abstract

**Purpose:**

To evaluate the pharmacokinetic properties of UGN-101, a mitomycin-containing reverse thermal gel used as primary chemoablative treatment for low-grade upper tract urothelial carcinoma (UTUC), in a subset of patients participating in a phase 3 clinical trial.

**Methods:**

Pharmacokinetic parameters (*C*_max_, *T*_max_, AUC_(0–6)_, *λz*, *t*_½_, and AUC_inf_) were evaluated in six participants (male or female, ≥ 18 years) with biopsy-proven, low-grade UTUC who received the first of 6 once-weekly instillations of UGN-101 to the renal pelvis and calyces via retrograde ureteral catheter. Plasma samples were collected prior to instillation and 30 min, 1, 2, 3, 4, 5, and 6 h post-instillation. Safety was assessed by laboratory evaluations, physical exam, and adverse event monitoring.

**Results:**

The mean age of the six participants was 69 years; most were male (5/6) and Caucasian (5/6). Mean (SD) *C*_max_ was 6.24 (4.11) ng/mL and mean *T*_max_ was 1.79 (1.89) hours after instillation. Mean apparent *t*_½_ following instillation was 1.27 (0.63) hours. Mean total systemic exposure to mitomycin up to 6 h post-instillation was 20.30 (19.69) ng h/mL. At 6 h post-instillation, mitomycin plasma concentrations of 5/6 participants were < 2 ng/mL. There were no clinically important adverse events or changes in laboratory values in any participant after a single instillation of UGN-101.

**Conclusion:**

The reverse thermal gel formulation of UGN-101 is associated with higher concentration and extended dwell time of mitomycin in contact with the urothelium of the upper urinary tract while limiting systemic absorption of mitomycin.

**Registration:**

NCT02793128; registered June 8, 2016.

## Introduction

Upper tract urothelial carcinoma (UTUC) is a rare malignancy, most commonly diagnosed in male patients > 70 years of age [1]. Current European Association of Urology (EAU) guidelines divide UTUC into low- and high-risk disease [2], with the goal of optimizing treatment based on both patient and tumor characteristics [3]. Radical nephroureterectomy (RNU) is considered the standard of care for treatment of organ-confined high-risk UTUC [3], but kidney-sparing surgery, such as endoscopic ablation, increasingly has been used for a subset of UTUC patients with low-grade, non-invasive disease manifesting as a small (< 20 mm), solitary, and favorably located lesion [2, 4]. Endoscopic management of UTUC, however, is associated with a high rate of tumor recurrence [4], and efforts to improve outcomes using intracavitary instillation of adjuvant chemotherapy have met with limited success [5], presumably due to the short dwell times achieved in the upper tract when using aqueous solutions that are continuously diluted by urine flow [4, 5]. Ultimately, most patients with low-grade UTUC undergo RNU [6, 7].

UGN-101 (JELMYTO^®^ [mitomycin] for pyelocalyceal solution) is a mitomycin-containing reverse thermal gel (4 mg mitomycin per mL gel) that has been developed as a primary chemoablative treatment for low-grade UTUC [8]. A slightly viscous liquid at room temperature, UGN-101 is instilled into the renal pelvis and calyces via ureteral catheter or nephrostomy tube where it becomes a semisolid gel depot at body temperature. Normal urine flow dissolves the gel over a period of several hours, allowing for increased drug concentration and dwell time at the site of tumor compared with aqueous solutions. Preclinical studies have demonstrated the feasibility and safety of serial retrograde instillations of UGN-101, and that intracavitary delivery of UGN-101 is associated with low levels of systemic mitomycin absorption [9–11].

In an early stage compassionate use protocol in severe, non-resectable UTUC [12] and in an open-label, single-arm, phase 3 clinical trial [8], UGN-101 treatment of patients with low-grade UTUC was associated with clinically significant and durable disease eradication, irrespective of baseline demographic or clinical characteristics, suggesting UGN-101 may provide a novel kidney-sparing alternative to patients with low-grade UTUC. Here we report results from a substudy of the phase 3 trial that evaluated the pharmacokinetic properties of instilled UGN-101 in six participants.

## Methods

### Subjects

Among participants who were enrolled in the OLYMPUS trial, an open-label, single-arm, phase 3 trial of UGN-101 conducted in the USA and Israel (NCT02793128), the first six who consented to participate in pharmacokinetic analyses were included in this substudy. Inclusion and exclusion criteria for the phase 3 trial of UGN-101 have been described extensively elsewhere [8]. Briefly, eligible participants were 18 years of age or older with primary or recurrent biopsy-proven, low-grade UTUC involving the renal pelvis and/or calyces. Participants were required to have life expectancy > 24 months, and an Eastern Cooperative Oncology Group performance status score < 3 (Karnofsky Performance Status score > 40), as well as adequate organ and bone marrow function, as determined by routine laboratory testing. Individuals who received Bacillus Calmette–Guérin treatment during the 6 months prior to the study and those being treated with systemic or intravesical chemotherapy were excluded.

The study was conducted in accordance with the International Conference on Harmonization Good Clinical Practice and the Declaration of Helsinki, and was approved by the designated ethics committees and institutional review boards at each site. All participants provided written informed consent.

### Drug dosage and treatment plan

Pharmacokinetic parameters were evaluated after participants received the first of 6 planned once-weekly instillations of UGN-101 to the renal pelvis and calyces via retrograde ureteral 5 or 7 French catheter. The instilled volume of UGN-101 was determined for each participant by averaging 3 fluoroscopically guided volumetric measurements of the renal pelvis and calyces before treatment and was not to exceed 15 mL (60 mg mitomycin).

### Blood sampling and preparation

Plasma samples were collected prior to instillation of UGN-101 and at 30 min, 1, 2, 3, 4, 5, and 6 h post-instillation. At each timepoint, 3 mL of blood was collected into K_2_EDTA tubes and placed immediately in ice, in a closed container protected from light. Using a refrigerated centrifuge, tubes were centrifuged within 1 h at 3000 RPM for 10 min to separate plasma, which was then collected using a disposable pipette into two 2 mL Eppendorf tubes labelled with the corresponding timepoint, placed in a box protected from light, and immediately stored at − 70 °C. One set of Eppendorf tubes was shipped to the central laboratory (AIT Bioscience Lab; Indianapolis, IN) for assessment, while the second set was retained at the local site as backup.

### Pharmacokinetic analyses

Pharmacokinetic analyses were performed by AIT Bioscience Lab using Bioanalytical Method BAM.0253.05 to assay study samples for the quantification of mitomycin in K_2_EDTA human plasma. The method is based on liquid–liquid extraction followed by liquid chromatography with tandem mass spectrometry instrumental analysis, and covers a measurement range from 0.100 to 100 ng/mL for mitomycin, using mitomycin C–^13^C–^15^N_2_ as the internal standard. Raw data from the mass spectrometer were acquired using Thermo Scientific (Waltham, MA) TSQ Module (v.1.0) and processed using Thermo Scientific Watson Laboratory Information System™ (v.7.4 SP4) for regression analysis and computation of sample concentrations.

Pharmacokinetic parameters that were assessed included maximum plasma concentration (*C*_max_), time to maximum plasma concentration (*T*_max_), area under the plasma concentration–time curve from 0 to 6 h (AUC_(0–6)_) with a concentration greater than or equal to the lower limit of quantification (LOQ, < 0.100 ng/mL), elimination rate constant (*λz*), terminal half-life (*t*_½_), and area under the plasma concentration–time curve extrapolated to infinity (AUC_inf_).

### Safety and toxicity analyses

Safety was assessed by laboratory evaluations, physical exam, and adverse event (AE) monitoring, as reported by the participant either spontaneously or in response to a non-leading question, or as observed by the treating clinician, and graded according to the Medical Dictionary for Regulatory Activities, version 19.1. Laboratory assessments included complete blood count, liver and renal function tests, coagulation, urinalysis, and urine culture. Blood tests were performed approximately 3 days prior to each instillation of UGN-101 to monitor the adequacy of organ and bone marrow function.

## Results

This pharmacokinetic substudy was conducted between April 6, 2017 and March 15, 2018, during which time the six participants enrolled in the study, received their first instillations of UGN-101, and had their blood samples collected, prepared, and shipped to the central laboratory where pharmacokinetic analyses were performed. The participants in the substudy were enrolled at Weill Cornell Medicine (*n* = 1), The Ohio State University (*n* = 1), the University of North Carolina (*n* = 1), and Sheba Medical Center (*n* = 3).

Baseline demographic and clinical characteristics of the substudy participants were generally similar to those reported for the overall population enrolled in the phase 3 trial [8] and are shown in Table 1. Most participants in the substudy were male (5/6) and Caucasian (5/6). The participants’ mean age was 69 years (median 68, range 62–81). All had a history of or current use of tobacco products. Although the protocol allowed for the presence of ≥ 1 low-grade lesion above the ureteropelvic junction measuring 5–15 mm in the greatest dimension, the participants in the substudy each had a single tumor, measuring ≤ 10 mm. The actual volume of UGN-101 delivered during the first instillation matched the planned volume for five of the six participants, while for participant 3 the actual volume instilled was 10 mL compared with a planned volume of 9.7 mL.

**Table 1:** Baseline demographic and clinical characteristics

Participant	Age	Sex	Race	BMI	History or current tobacco use	Number of kidneys	Papillary tumor burden			Volume of renal pelvis and calyces (mL)
							Number	Diameter of largest (mm)	Total burden (mm)	
1	73	F	Caucasian	17.0	Yes	2	1	10	10	7.0
2	67	M	Caucasian	32.8	Yes	1-R	1	10	10	10.0
3	62	M	African American	22.9	Yes	2	1	8	8	9.7
4	69	M	Caucasian	30.4	Yes	2	1	8	8	12.0
5	81	M	Caucasian	28.9	Yes	2	1	5	5	9.7
6	63	M	Caucasian	20.9	Yes	2	1	5	5	11.0

*BMI* body mass index, *F* female, *M* male

UGN-101 pharmacokinetic parameters for each of the six participants are shown in Table 2. At 6 h post-instillation, the mitomycin plasma concentrations of five of six participants were < 2 ng/mL, with the plasma concentration of one participant dropping below the LOQ. Mean *C*_max_ was 6.24 ng/mL (range 2.43–12.80 ng/mL), and mean *T*_max_ was 1.79 h (range 0.50–5.17 h) after instillation. The mean apparent *t*_½_ following instillation of UGN-101 was 1.27 h (76 min). The mean total systemic exposure to mitomycin up to 6 h post-instillation, AUC_(0–6)_, was 20.30 ng h/mL (range 5.64–58.76 ng h/mL). One subject (participant 6) did not exhibit a terminal log-linear phase in the concentration–time data; therefore, no values for AUC_inf_, *λz*, or *t*_½_ are reported for this subject.

**Table 2:** Summary of pharmacokinetic parameters following instillation of UGN-101

Participant	*C*_max_ (ng/mL)	*T*_max_ (h)	AUC_(0–6)_ (ng h/mL)	*λz* (*h* − 1)	*t*_½_ (h)	AUC_inf_ (ng h/mL)
1	2.43	2.92	10.72	0.30	2.28	14.79
2	9.47	0.98	23.10	0.49	1.42	24.60
3	3.53	0.50	5.64	1.03	0.67	5.80
4	3.22	0.58	11.82	0.83	0.84	12.17
5	5.97	0.58	11.78	0.60	1.16	12.09
6	12.80	5.17	58.76			
Mean (SD)	6.24 (4.11)	1.79 (1.89)	20.30 (19.69)	0.65 (0.28)	1.27 (0.63)	13.89 (6.84)
Geometric mean (CV)	5.21 (0.73)	1.17 (1.26)	15.01 (0.95)	0.60 (0.50)	1.16 (0.50)	12.54 (0.56)

*C*_max_ is the highest concentration achieved in plasma

*T*_max_ is time to highest plasma concentration

AUC_(0–6)_ is the area under the plasma concentration–time curve from zero to 6 h

AUC_inf_ is the area under the plasma concentration–time curve from zero to extrapolated infinity

*λz* is elimination rate constant

*t*_½_ is terminal half-life

Participant 6 did not exhibit a terminal log-linear phase in the concentration–time data; therefore, no values for *λz*, *t*_½_, or AUC_inf_ are reported

*CV* coefficient of variation, *SD* standard deviation

**Fig. 1:**
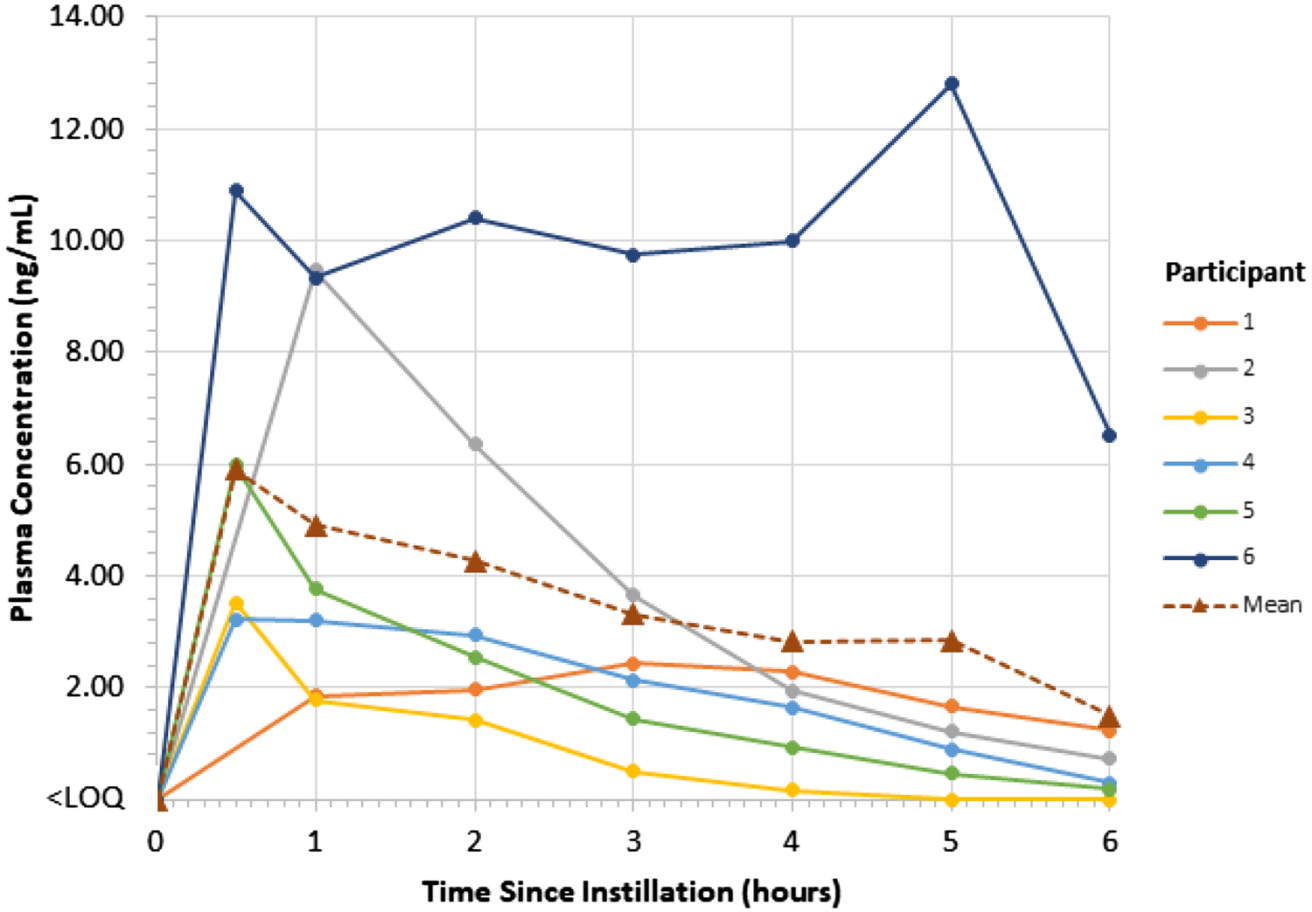
Mitomycin concentration vs time curve following instillation of UGN-101. LOQ, limit of quantification

The mitomycin plasma concentration vs. time curve for each participant following instillation of UGN-101 is shown in Fig. 1. Mean (SD) mitomycin concentration in plasma was below the LOQ pre-instillation and 5.91 (3.55) ng/mL, 4.90 (3.57) ng/mL, 4.27 (3.47) ng/mL, 3.32 (3.32) ng/mL, 2.83 (3.60) ng/mL, 2.84 (4.91) ng/mL, and 1.49 (2.50) ng/mL at 30 min, 1, 2, 3, 4, 5, and 6 h post-instillation, respectively. The median (range) mitomycin concentration in plasma was 0.51 (0.00, 6.52) ng/mL at 6 h post-instillation.

There were no clinically important adverse events or changes in laboratory values after the first instillation of UGN-101 in the substudy participants. Mild hyperkalemia that was considered unrelated to treatment occurred in a single patient (participant 2) 7 days after exposure, and was the only adverse event reported among the six patients during the substudy. Mild leukopenia (observed as a lymphocyte count equaling 20% of leukocytes) was reported in 1 patient (participant 2) after 3 instillations of UGN-101 (7 days after most recent exposure) but was not considered clinically significant and resolved within one week. Two patients experienced ureteral stenosis that was considered related to the study drug. In one patient (participant 5), stenosis occurred after 14 instillations of UGN-101, and was considered mild in severity. In the other (participant 6), moderate stenosis occurred after 6 instillations of UGN-101, and recurred after 8 and 10 instillations, leading to hospitalization. In this same patient, clinically significant increased creatinine level and reduced glomerular filtration rate were reported after 11 instillations of UGN-101. No other changes in laboratory values were considered clinically significant in the substudy participants.

## Discussion

A subset of patients in a phase 3 study of UGN-101 for the treatment of low-grade UTUC consented to a study of systemic mitomycin exposure by providing blood samples for pharmacokinetic analysis, to elucidate plasma levels and time course of drug exposure. It was anticipated that instillation of UGN-101 via retrograde ureteral catheter to the renal pelvis and calyces would result in low systemic exposure to mitomycin while realizing prolonged exposure to the chemotherapeutic agent in the target organ, thereby mitigating the drug’s known myelosuppressive toxicity risks while achieving maximal therapeutic benefit.

Analysis of individual participant plasma concentration versus time profiles showed that at 6 h post-instillation, systemic exposure to mitomycin was limited. Mitomycin plasma concentrations in five of six participants were below 2 ng/mL, with the plasma concentration of one participant dropping below the LOQ. The highest observed *C*_max_ value in a participant was 12.8 ng/mL, which is 187-fold and 40-fold lower than observed *C*_max_ levels following an intravenous bolus dose of 30 mg or 10 mg mitomycin (2.4 μg/mL and 0.52 μg/mL, respectively) [13], doses that typically would be used for the treatment of disseminated adenocarcinoma of the stomach or pancreas in combination with other chemotherapeutic agents. The highest observed *C*_max_ value is 31-fold lower than the 400 ng/mL threshold for myelosuppression observed with mitomycin [14].

The mean apparent *t*_½_ following instillation of UGN-101 was 1.27 h (76 min), which is longer than the mean *t*_½_ of approximately 17 min following a bolus injection of 30 mg mitomycin [13]. The apparent *t*_½_ suggests that UGN-101 disintegrated gradually, resulting in prolonged exposure of the target organ to mitomycin following local instillation of UGN-101 into the upper urinary tract. In vitro models, in vivo studies, clinical trials, and computer simulations have all shown that the efficacy of mitomycin improves with increasing drug concentration and exposure time at the target site [15–23].

No clinically significant adverse events or meaningful changes in laboratory values were observed among participants in this study within 7 days of the first instillation of UGN-101, although one subject (participant 2) experienced mild leukopenia after several instillations of UGN-101 that may have been related to mitomycin exposure. A second subject (participant 6)—whose total systemic exposure to mitomycin was 2.5–10 times greater than other participants in the substudy—experienced moderate to severe ureteral stenosis after 8 and 10 instillations of UGN-101, and clinically significant changes in creatinine level and glomerular filtration rate after 11 instillations of UGN-101 (6 weekly infusions followed by monthly maintenance infusions), that may have been related to treatment. Detailed discussion of treatment-related morbidity that may occur as a result of multiple instillations of UGN-101 for the management of low-grade UTUC has been presented previously [8].

There are limitations to the current study. First, there were a small number of participants, of which five of six were male and five of six were Caucasian, potentially limiting the generalizability of the findings. However, UTUC is a rare cancer, occurring with an estimated annual incidence of approximately 2 per 100,000 individuals in Western countries [24], and is three times more common in men than in women [3]. Second, pharmacokinetic parameters were assessed after the initial instillation of UGN-101, whereas the treatment protocol for low-grade UTUC called for 6 weekly-instillations of UGN-101 as induction therapy followed by up to 11 monthly maintenance treatments [8]. Analyses of mitomycin pharmacokinetics following multiple instillations of UGN-101 and in larger patient populations should be a focus for future study.

In conclusion, a phase 3 trial has shown that primary chemoablation of low-grade UTUC with intracavitary UGN-101 results in clinically significant disease eradication, irrespective of baseline demographic and clinical characteristics, and may offer a novel kidney-sparing treatment alternative for these patients, particularly those with tumors that are difficult to treat endoscopically [8]. The current pharmacokinetic substudy of the phase 3 trial has demonstrated that the UGN-101 reverse thermal gel formulation achieves increased concentration and extended dwell time of mitomycin with the surface of the upper urinary tract while limiting systemic absorption of mitomycin to levels well below known toxicity thresholds.

## Data Availability

The study protocol is available as an online appendix to Kleinmann N, Matin SF, Pierorazio PM, Gore JL, Shabsigh A, Hu B et al. Primary chemoablation of low-grade upper tract urothelial carcinoma using UGN-101, a mitomycin-containing reverse thermal gel (OLYMPUS): an open-label, single-arm, phase 3 trial. *Lancet Oncol*. 2020;21(6):776–85. doi:https://doi.org/10.1016/S1470-2045(20)30147-9. Individual participant data that underlie the reported results are not available.
